# Selecting the Acceptance Criteria of Medicines in the Reimbursement List of Public Health Insurance of Iran, Using the “Borda” Method: a Pilot Study

**Published:** 2015

**Authors:** Amir Viyanchi, Hamid Reza Rasekh, Ali Rajabzadeh Ghatari, Hamid Reza SafiKhani

**Affiliations:** a *School of Pharmacy, Shahid Beheshti University of Medical Sciences, Tehran, Iran.*; b *Tarbiat Modares University, Tehran, Iran. *; c *Member of Strategic Council at National Research Network for Policy Making, Economics and Health Management, Tehran, Iran.*

**Keywords:** Health Insurances, Decision Making, “Borda” Method, Medicine Reimbursement

## Abstract

Decision-making for medicines to be accepted in Iran’s public health insurance reimbursement list is a complex process and involves factors, which should be considered in applying a coverage for medicine costs. These processes and factors are not wholly assessed, while assessment of these factors is an essential need for getting a transparent and evidence-based approach toward medicine reimbursement in Iran.

This paper aims to show an evidence-based approach toward medicine selection criteria to inform the medical reimbursement decision makers in Iranian health insurance organizations.

To explore an adaptable decision-making framework while incorporating a method called “Borda” in medicine reimbursement assessment, we used the help of an expert group including decision makers and clinical researchers who are also policy makers to appraise the five chief criteria that have three sub criteria (Precision, Interpretability, and Cost). Also software “Math-lab”7, “SPSS” 17 and Excel 2007 were used in this study.

“Borda” estimates the amount of perceived values from different criteria and creates a range from one to five while providing a comprehensive measurement of a large spectrum of criteria. Participants reported that the framework provided an efficient approach to systematic consideration in a pragmatic format consisting of many parts to guide decision-makings, including criteria and value (a model with the core of Borda) and evidences (medicine reimbursement based on criteria).

The most important criterion for medicine acceptance in health insurance companies, in Iran, is the "life-threatening" factor and "evidence quality" is accounted as the fifth important factor. This pilot study showed the usefulness of incorporating Borda in medicine reimbursement decisions to support a transparent and systematic appraisal of health insurance companies' deeds. Further research is needed to advance Borda-based approaches that are effective on health insurance decision making.

## Introduction

Decision-making for adding a new drug to the medicine reimbursement list of Iranian third-party payers (Insurers) is a complex process; so efficient and explicit processes to ensure transparency and consistency in considered factors are required. Also, the necessity to use clinical evidence and information regarding medicines coverage in decision making process of health insurance organizations is not really clear. There is a compelling need for a transparent and evidence-informed approach toward medicine reimbursement in Iran. Such an approach to show the relative advantages of medicine criteria selection, in order to inform reimbursement decision makers should be the goal.

Every year, more effective medicines are produced to deal with a large number of diseases. The availability of so many medicines raises this question: how can insurers provide their insured with access to essential medications, and meanwhile control the cost of drugs? As evidence-based medicine has become ubiquitous in clinical decision-making discussions, conflicts over definitions and proper application of evidences are a decade-old problem and remain controversial in medical necessities debates (1, 2, 3).

Medicines with high benefits and low costs are the ideal drugs for insurers seeking to improve the health of the insured while managing drug costs; however, decision making in some situations is complicated. For instance, it should be investigated whether the cost of an effective and beneficial medicine is considered. Several beneficial methods are proposed and carried out by diverse countries such as Canada, China, Australia, Germany, and the United Kingdom. Methods including reference pricing (4, 5), generic substitution (6), income-based deductibles (7), co-payments and coinsurance (8, 9), incentive-based tiered formularies (10), negative and positive subsidy lists (11), prescribing budgets (10, 12), and drug caps (13), and each of them has led to varied results. In decision making for coverage, decision makers may face a dilemma between their interests in cost analyses and fear of public and even a professional backlash. General spending on health care are not immune to the pressures of political and social powers, which are a mixture of the existing injustice (14).

In this paper, we explain an approach of decision making on medicine reimbursement for Iran’s health insurance organization by taking advantage of the “Borda” method, in a pilot study.

**Figure 1:**
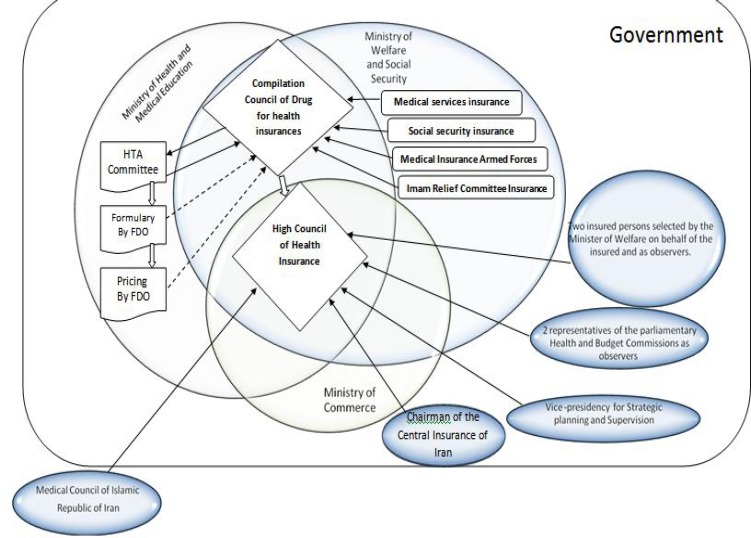
Relations of reimbursement decision maker bodies for new medicine selection in Iran health insurances

**Figure 2:**
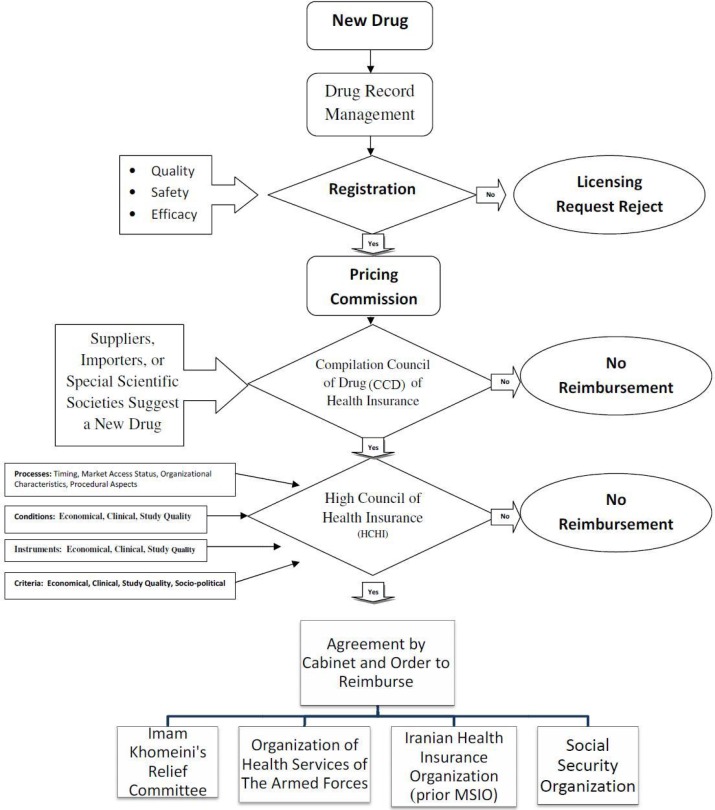
Process of reimbursement decision making for new medicine selection in Iranian public health based insurance organizations

Decision-making organizations in Iran who are responsible for new medicines reimbursement (third-party payers) are various and multi-segmented. The details of these relationships and their impact on novel medicines reimbursement are shown in Figure 1. Also, the reimbursement process for new medicines that takes place by health insurance organizations in Iran is depicted in Figure 2. As shown in the Figure, the Compilation Council of Drug (CCD) of health insurances plays a significant role in the acceptance of an offer for new medicines that are usually generic medicines, and evaluates its characteristics using the information collected from Iranian drug-making companies or drug-importing firms. In this regard, information such as clinical benefits, clinical documentation, and guidelines and so on are not wholly clarified and stabled as they should be. Then, the council grants its recommendation to the High/superior Council of Health Insurance (HCHI). This superior council consists of members from ministry of health and medical education, ministry of labor, cooperation and social welfare, ministry of commerce, vice-president for strategic planning and supervision and medical council of the Islamic Republic of Iran (non-governmental). If the council agrees with the novel medicine’s reimbursement, they notify all Iranian health insurance organizations.

**Figure 3.**
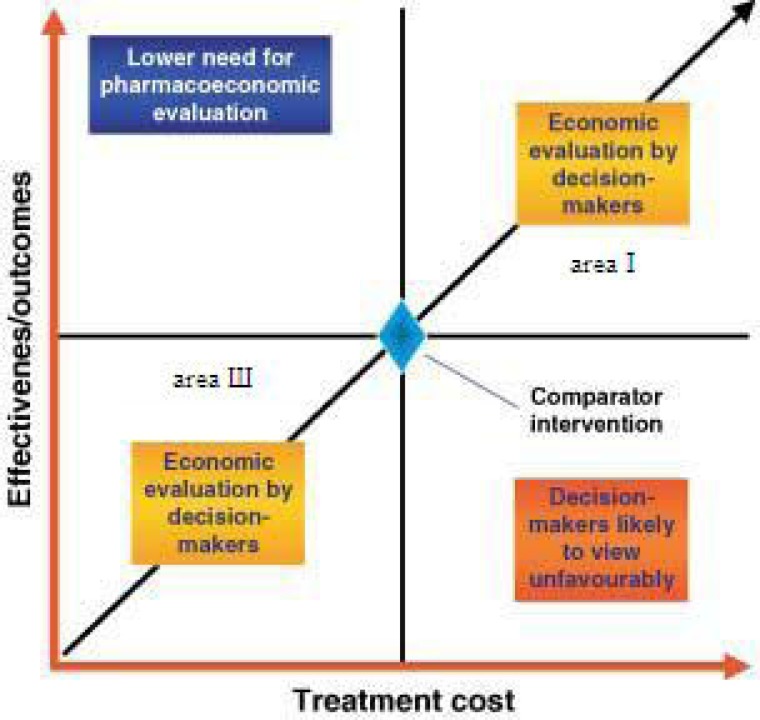
How decisions analyses are made?

How decisions and analyses are made? Guzman believed the main parts (15) that must be considered in any decision evaluation are (Figure 3):

1. Perspective (health trusts, governmental body, insurance companies, patients and society);
2. Time horizon;
3. Costs (direct medical costs, direct non-medical costs, indirect costs, intangible costs); and
4. Outcome (years of life saved, years of disease-free survival, cure rate).

Adopted from: R Arenas-Guzman.2005, P: 35.

We will face the trouble of decision making when the costs and benefits of a new medication are both high and low (areas Ι and Ш in Figure 3). Time and perspective affect the cost and benefits of medicines and in some cases, if there is valid quantitative evidence we can judge by it. When we do not have the adequate documentation, collection of experts and elites’ opinions, who have enough experience in the related area, is required.

**Figure 4:**
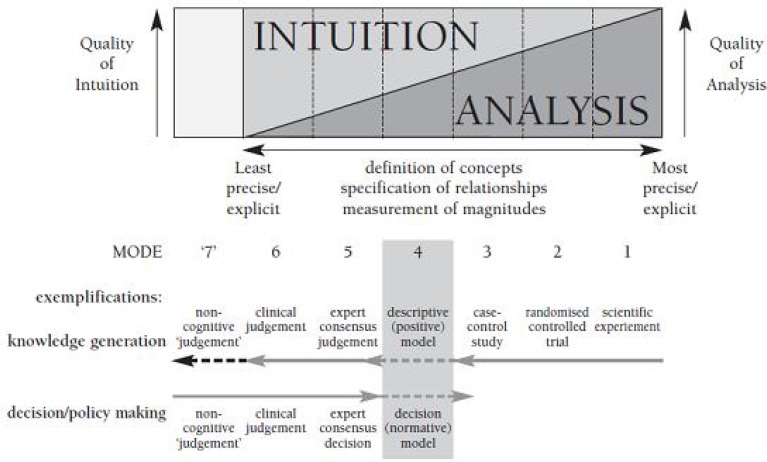
Measurement of magnitudes for modes of decision-making, Adopted from: Jack Dowie, Health Care Priority Setting, 2003, p: 10

As shown in Figure 4 (cognitive dimensions of cognitive chain framework), analysis accuracy of the experts’ consensus judgment is moderate and it is more based on intuition than scientific analysis. The absence of much needed evidence, leads us to modes 5 and 6.

In this article, decision-making is done at mode 5 (taking into account the previously mentioned points) (16).

A drawback of simple methods is the risk of low discriminatory power. Many other methods those weight ideas differently (for example, analytical hierarchy process (AHP); multi attribute utility analysis; swing weighting and conjoint analysis) that have varying degrees of complexity (17, 18).

Structure of research material collection is organized as 4 steps follow:

Step one: Extraction and classification of different criteria and first questionnaire construction. 

26 effective criteria for medicine selection in the health insurance were extracted from the literature and articles from developed countries. Acquired criteria were categorized in 4 parts. 

The first part, economic criterion, consisted of 7 criteria. The second part, clinical criterion, also consisted of 7 criteria. The third part, study quality criteria and finally the forth part, management criterion, both include 6 criteria. For data gathering a questionnaire was developed with 26 questions about criteria (Table 1). Its reliability evaluation according to Cronbach's alpha was 96%.

**Table 1:** Different parts of extracted criteria for decision-making assessment.

Part	Criteria	Reference
1-economical	1-economic evaluation guidelines by manufacturer 2-ICER thresholds 3-Budget impact 4-Expected budget increase for third party payer 5-Price in comparison to comparator drug 6-Sales volume of drug 7-Physicians demand	(19,20) (21-25) (21,26,27) (28,29) (30,31) (32) (33)
2- clinical	1-Considered RCT evidence 2-DALY as an endpoint of drug 3-Efficacy as a therapeutic value 4-Availability of treatment alternative 5-Condition is life threatening 6-Target population 7-Ability to reduce own health risk	(19,21,24,28,34,35) (24,34) (30,36,37) (21,24,26,38) (28,36,38) (24) (24)
3-study quality	1-Quality of evidence 2-Quality of economic model 3-Quality of economic evidence review of stakeholder perspectives 4-Degree of uncertainty 5-Consideration of HTA 6-Year of study publication	(27,39) (24,28,35,40) (24,28,35,40) (24,28,35,40) (26,28,38) (19,41) (24)
4-management field	1-rule of rescue 2-Ensure the availability of drugs 3-Place in therapeutic strategy (e.g. 1st or 2nd line treatment) 4-Objective of technology (e.g. prevention) 5-Objective of technology (e.g. treatment) 6-Expert Committee Decision	(42) (32) (36) (24,38) (24,38) (43,44)

Step two: Determining the highest score (mean/SD) in each part using the feedbacks of drug and insurance experts. Responders included health insurance industry specialists and medical experts (purposed sampling). Measurement tool was a scale from 1 to 100 with 1 representing the least and 100 the most important criteria and each respondent was asked to mark the criterion importance on it. From 80 respondents, 45 questionnaires returned (Table 2). Also we used the software “SPSS” 17 and Excel 2007.

In this step, 5 criteria reached the highest rank including:

A1-Expert Committee Decision

A2- Life- threatening condition

A3-Quality of evidence

A4- RCT evidence consideration

A5-Economic evaluation by manufacturer, (Table 3)

**Table 2:** Demographic specifications of responders to questionnaire

Variable	Range	n	
Gender	Male Female	28 17	
Age	<40 =>40, <=45 >46, <=50 >50	15 18 8 4	
Education	Master MD PhD	3 26 16	
Cross study	Physician Pharmacy Medical sciences	7 31 7	
Work experiment(years)	O <3 >=3,<=10 >=11,<=15 >=16,<=20 >20	2 4 7 9 12 11	
Health insurance compilation council of Drug experiment (years)	O <3 >=3,<=10 >=11,<=15	27 7 9 2
High council of health insurance experiment(years)	O <3 >=3,<=10 >=11,<=15	28 8 7 2

**Table 3:** Primary ranking of preferred criteria by expert responders

Rank	Criteria	Score(mean/SD)
1	Expert Committee Decision	5.763
2	Condition is life- threatening	5.405
3	Quality of evidence	4.831
4	Considered RCT evidence	4.484
5	economic evaluation guidelines by manufacturer	4.297

Step Three: Choosing 3 sub-criteria (x_1_: Precision, x_2_: Interpretability and x_3_: Cost) (45) that were rated by Delphi out of 8 sub-criteria, and advisers defined the weights in this order:

$\left(w_{1}=0.2,w_{2}=0.3,w_{3}=0.5\right)$

Then, we evaluated the 5 residual criteria under three sub-criteria in second questionnaire which planed on a 5 point LIKERT scale (1–very little 2–little 3–medium 4–much 5–very much).

Step Four: Determining the weight of 5 selected criteria in the second step using three sub-criteria selected in the third step by 6 elite members (Table 4) of Iran’s Food and Drug organization (2 person), health insurance organization (2 person) and the University of Tehran’s Medical Ethics Department (2 person). These 6 experts were asked to weight the five main criteria from 1 to 5 while taking into account the 3 sub-criteria. 6 matrices were created and the results were obtained using the *Borda* method and Mat-lab7 software.

**Table 4:** Demographic specifications of 6 experts

Variable	Range	n	
Gender	Male Female	5 1	
Age	=>40, <=45 >46, <=50 >50	1 2 3	
Education	MD PhD	4 2	
Field of education	Physician Pharmacy	2 4	
Work experiment(years)	>=11,<=15 >=16,<=20 >20	1 1 4	
Experiment work in Health insurance compilation Council of Drug (years)	O >=3,<=10 >=11,<=15	4 1 1
Experiment work in High council of health insurance (years)	O >=3,<=10	4 2

MCDM models are used in different researches recently and the Borda rule, a well-known and appropriate group decision- making procedure was originally proposed for linear orders in Borda (46) in what follows, we will call it classic Borda rule. It has been widespread analyzed and extended to more general orders from its initial design (47, 48). In the last years it has also been considered in a fuzzy framework (49- 52), and in a linguistic context (53).

“Borda” method prioritizes “m” options, against “n” indicators (quantitative and qualitative); by “k” decision-makers while using ordinal scale. Assuming a group of decision maker (DM) reach a consensus on common indicators, at first each DM ranks the indicators) D^P^) from the available options in the direction of the target of the issue, then analysis takes place according to the following steps:

1- Ratings results of all “k” decision makers, for j^th^ indicator, forms the matrix Rj, So, "n" Matrix, for each" n" indicators, will be given.

2- Ranking of each matrix R_j_ for every indicator j^th^, converted to a Borda number for each decision maker p^th^ (among the “k” decision makers). In the way that this option is ranked first by the decision maker p^th^ has “m-1” relative value. The second option’s relative value is “m-2”, and option with the rank of “m^th^” will have relative value of “0”. In this way, Prof. “Borda” transformed a ratio scale to some kind of relative scale and made the additive practice possible.

3- Row sum up of the matrix B_j _(“Borda” numbers), for "k" decision makers $\left(\sum_{p=1}^{k}b_{i,j}^{p}\right)$ will be obtained and we will calculate the final ranking of each option for each indicator j^th^ so that the row with maximum sum of Borda numbers will have the first rank and the row with minimum summation will get the m^th^ rank (if a node was created, we’d open it properly). 

$r_{i,j}^{'}$ of this matrix represents the rank of group consensus for the option i^th^ for indicator j^th^. 

1- As following, indicator weights (w_j_) are calculated appropriately, using SWING methods and then a weighted matrix of group consensus, with m rank of “m” option.

The parts of this matrix are $q_{i,t}=\sum_{j=1}^{n}\pi_{\mathrm{iti}}.w_{j}$ , so that $\pi_{\mathrm{itj}}=1$ , if the option i^th^ in rank t^th^ return to indicator j^th^, and otherwise will be zero.

1- The following assignment-problem with variables of h_it _= {0, 1} must be solved to determine the final ranking of criteria.

In the final solution, h_it_ = 1, if the rank of t^th^ has been allocated to the option i^th^; otherwise, it is equal to zero. 

## Results

Five criteria for medicine reimbursement (m = 5) for each sub-criterion (n = 3) are ranked by a group of decision makers consisting of 6 medicine experts (K = 6).

1- All the six experts’ judgments, for each sub-criterion, in one of the three matrices R_j_ (With 3 sub-criteria) are summarized.

2- Matrix “Borda” (B_j_) for each sub-criterion x_j_ is formed. 

3- Row sum up of the matrix B_j_j is calculated, and we get the final grade (marginal rank) through the group consensus for each option for each indicator j^th^.

4- As for the weights of indicators we have: $W=(w_{1}=0.2,w_{2}=0.3,w_{3}=0.5)$

) 

Thus, for the weighted matrix from the group consensus of (Q_G_) regarding $q_{i,t}=\sum_{j=1}^{3}\pi_{\mathrm{iti}}.w_{j}$ .When substituting the values of w_j_, Q_G_ is appeared.

5- Final prioritizing the criteria through solving zero and one as the above (using the Hungarian method for matrix Q_G_) is as follows:


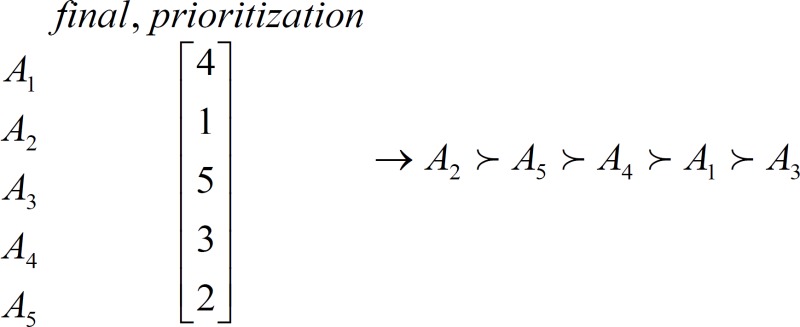


The *Borda *method helped to find the criterion with the highest importance for admission of medicine for third-party payers in Iran, which was the life-threatening factor, and the fifth rank was assigned to the evidence quality criterion (Table 5).

**Table 5:** secondary ranking from preferred criteria by”Borda” method

Rank	Criteria	Symbol
1	Condition is life- threatening	A_2_
2	Economic evaluation guidelines by manufacturer	A_5_
3	Considered RCT evidence	A_4_
4	Expert Committee Decision	A_1_
5	Quality of evidence	A_3_

## Discussion

1- Life-threatening condition is one of the factors that should always be considered by health insurers when they are deciding to add a specific drug to the reimbursement list. As it was found in this study, the most important criterion for admission should be the severity of disease. It is also confirmed in the study of Harris AH *et al*., Le Pen C and Clement:

An investigation to analyze the relative influence of various factors on decisions made for public insurance coverage of new drugs in Australia was performed by Harris AH and others. Clinical significance, cost-effectiveness, costs for the government, and severity of disease had significant influences on decision-makings. Compared to the average submission, clinical significance rose the probability of recommending coverage by 0.21 (95% confidence interval (CI) 0.02 to 0.40), but a drug in a life-threatening condition had a higher probability of being recommended for coverage of 0.38 (0.06 to 0.69) (29). 

Le Pen C and others with collecting data on the results of "medical service rendered" (MSR) used several criteria to perform a classification (efficacy, security, severity of the disease, being in a therapeutic strategy, existence of an alternative therapy, having the value of public hygiene) for a sample of 1453 drugs belonging to five therapeutic areas. Only two criteria -efficacy and disease severity- sufficed the adequate explanation of MSR classification and the other criteria had little value (37).

Also, Fiona M. Clement and others considered socially relevant characteristics of a patient group (*i.e.*, unmet needs in disadvantaged populations, severity of the condition (such as, life-threatening)) (39).

But, saving any patient's life should not be delayed because of long studies and decision makings.

2- The next criterion is pharmacoeconomic evaluation. Although this issue is not yet completely addressed and clear in Iran, pharmaceutical companies (whether manufacturer or importer) should be required to prove that their newly proposed drugs are economical. In articles of Neumann (2008) (20) and Titlow (2000) (21) following the economic evaluation guidelines, are emphasized a lot.

3- The third criterion is RCTs documentation. One of the most important tools to prove a drugs’ efficacy is accurate RCTs. Every year, many clinical trials are carried out in academic and research centers of Iran. Their results are used for medicine selection in the health insurance list and the accuracy of RCTs’ information gets reviewed in several references. Also, there is a RCT registration center in Iran, which is verified by the World Health Organization and all researchers are mandated to register their clinical trials on it (www.Irct.ir). The importance of this criterion has been emphasized in several papers (20, 22, 25, 29, 35, and 36) and it has called “the golden standard.”

4- The fourth criterion is to use the decisions of drug selection expert committees. As for now, 2 committees of HTA and Clinical studies exist in Iran. The HTA committee has been established recently and its ideas and comments are effective scientific resources for new medicine reimbursement decisions in Iranian public health insurance companies.

The committee of Clinical studies of Food and Drug Organization (FDO) started its activities in 2001. Its action began by the Iranian Ministry of Health order, after Medical Education following recognition good clinical practice (GCP) proposed by the World Health Organization. This committee is closely associated with the performances of the national committee for ethics in medical research and established for RCTs registration as the deputy of research and technology (www.hbi.ir(and the central scientific committee (www.irct.ir).

Secretariat of the Committee for Clinical Studies is a subsidiary office of medicines supervision and drugs (Narcotics), and its accomplishments include assessment of protocols, reports and files about clinical trials of medicines that the applicant's companies' request for diffusion or registration to the pharmaceutical market and the reimbursement list of public third party payers.

The secretariat of the Committee acts as the executive force of clinical studies committee which works with outsourcing mechanisms. Members of the clinical studies committee are composed of faculty members of universities of medical sciences, headquarters of the ministry and specialists of food and drug deputy that are established by the general director. Also, a network of over 90 clinical and academic experts in various fields, cooperate with the Secretariat as referees and controllers of clinical trials.

Even though there is no pharmacoeconomic scientific committee in Iran, like many other developing countries, there is a shortage of skilled pharmacoeconomists. Forming this committee along with other specialized committees for drug selection can mitigate the financial and economic burden of public health insurances.

According to Rogowski WH and other studies: "The comprehensiveness and relevance of this framework was assessed by an independent group of experts in HTA. Coverage decisions require medical, economic and legal expertise. Thus, they are usually made by interdisciplinary committees. For the German statutory health insurance, decisions are made on behalf of the Federal Associations of Sickness Fund, Physicians or the German Hospital Association and the Federal Associations of Sickness Funds by the G-BA. For the English National Health Service, NICE commission appraisal committees consisting of representatives of relevant stakeholders in the health care system to provide guidance on the technology under investigation. For the decisions on ACI made in the USA, the insurers' medical directors play a major role. Frequently, they rely on the support of an interdisciplinary committee of researchers and other independent experts." (44)

5- Evidence quality is the last criterion, which got through Borda, and states that we ought to be certain of the suitability of the way to take in information and data integrity at the time of deciding. In this regard, before conducting any study, we should evaluate the quality of previous studies. To do this, we used checklists and because evaluating studies’ quality is discretionary, more than one individual should be involved in the work of grading the studies. Mason AR, Drummond MF (40) and Chim L, *et al.* (28) in their article, have pointed to the importance of quality of studies regarding the reimbursement of expensive anti-cancer medicines.

Finally, we must pay attention to role of the sub criteria. Among the sub criteria mentioned above, the weight of the COST was more important in determining the rankings of main criteria other than sub criteria. It seems that the issue for determining the costs of criteria from the viewpoint of expert opinion is very important and selection the drug criterion should not be costly by itself. Sub criteria such as the Interpretability and Precision, respectively, have their own effect, and caused to change the initial ranking of the main criteria.

## Conclusion

The most important criterion for acceptance of medicines in the health insurance in Iran is the life-threatening condition of the patient and using quality evidence is ranked fifth. This pilot study showed the usefulness of incorporating “Borda” in the medicine reimbursement issue to support a transparent and systematic appraisal of health insurance interventions. Medicine selection for health insurance reimbursement is better to be an evidence-based approach as much as possible. Since the acceptable documents are not always available, sometimes there is a need to seek for experts’ opinions in the process of decision-making.

Based on the results obtained by Borda deciding method, it is clear that the sub criteria also play a sizable role in calculations of the results with different initial rankings (compare Tables 3 and 5). This pilot study is based on elites and experts’ intuitive decision-makings; therefore, we should consider some sub criteria and indicators, which weight the main criteria as well, because direct results that are only based on the average scores of main indicators may not be entirely precise.

Further research is needed to improve Borda-based approaches for a more effective decision making in the health insurance field and further experience is needed to make a better judgment about applying the Borda method. Also, we recommend comparing the accurateness and the criteria rankings to show whether it will provide a basis for more formal comparison of different criteria and determine their appropriateness for particular decision contexts. 
